# The clone devaluation effect: A new uncanny phenomenon concerning facial identity

**DOI:** 10.1371/journal.pone.0254396

**Published:** 2021-07-13

**Authors:** Fumiya Yonemitsu, Kyoshiro Sasaki, Akihiko Gobara, Yuki Yamada

**Affiliations:** 1 Graduate School of Human-Environment Studies, Kyushu University, Fukuoka, Fukuoka, Japan; 2 Japan Society for the Promotion of Science, Chiyoda-ku, Tokyo, Japan; 3 Faculty of Informatics, Kansai University, Takatsuki, Osaka, Japan; 4 Faculty of Arts and Science, Kyushu University, Fukuoka, Fukuoka, Japan; 5 BKC Research Organization of Social Science, Ritsumeikan University, Kusatsu, Shiga, Japan; Polish Academy of Sciences, POLAND

## Abstract

Technological advances in robotics have already produced robots that are indistinguishable from human beings. This technology is overcoming the uncanny valley, which refers to the unpleasant feelings that arise from humanoid robots that are similar in appearance to real humans to some extent. If humanoid robots with the same appearance are mass-produced and become commonplace, we may encounter circumstances in which people or human-like products have faces with the exact same appearance in the future. This leads to the following question: what impressions do clones elicit? To respond to this question, we examined what impressions images of people with the same face (clone images) induce. In the six studies we conducted, we consistently reported that clone images elicited higher eeriness than individuals with different faces; we named this new phenomenon the *clone devaluation effect*. We found that the clone devaluation effect reflected the perceived improbability of facial duplication. Moreover, this phenomenon was related to distinguishableness of each face, the duplication of identity, the background scene in observing clone faces, and avoidance reactions based on disgust sensitivity. These findings suggest that the clone devaluation effect is a product of multiple processes related to memory, emotion, and face recognition systems.

## Introduction

Artificial Intelligence (AI) technology and robotics have been improved at a remarkable and rapid pace. In recent years, the abilities of AI and robots have become equal to or have exceeded those of human beings. For example, AlphaGo, a computer Go program developed by Google DeepMind, has defeated human players at the game [1]. Pepper, an AI robot developed by SoftBank, can read human emotions from facial expressions and vocal tones [2].

Moreover, the abilities as well as the appearance of robots is becoming more human-like. However, people may feel unpleasant when encountering humanoid robots the appearance of which is similar to a real human to some extent (i.e., Uncanny valley [3]). Previous research on the uncanny valley phenomenon revealed that it occurs not only in response to humanoid robots [e.g. 4] but also to computer graphic (CG) characters [5] and can prevent people from comfortably viewing animated works and interacting with androids. Although it was very difficult to produce robots that look exactly like human beings when Mori proposed the uncanny valley, today technology has been developed enough to create closely human-like objects like Geminoid [6, 7] and Saya [8]. Thus, technological advances may be overcoming the uncanny valley by developing robots that are indistinguishable from human beings.

**Fig 1:**
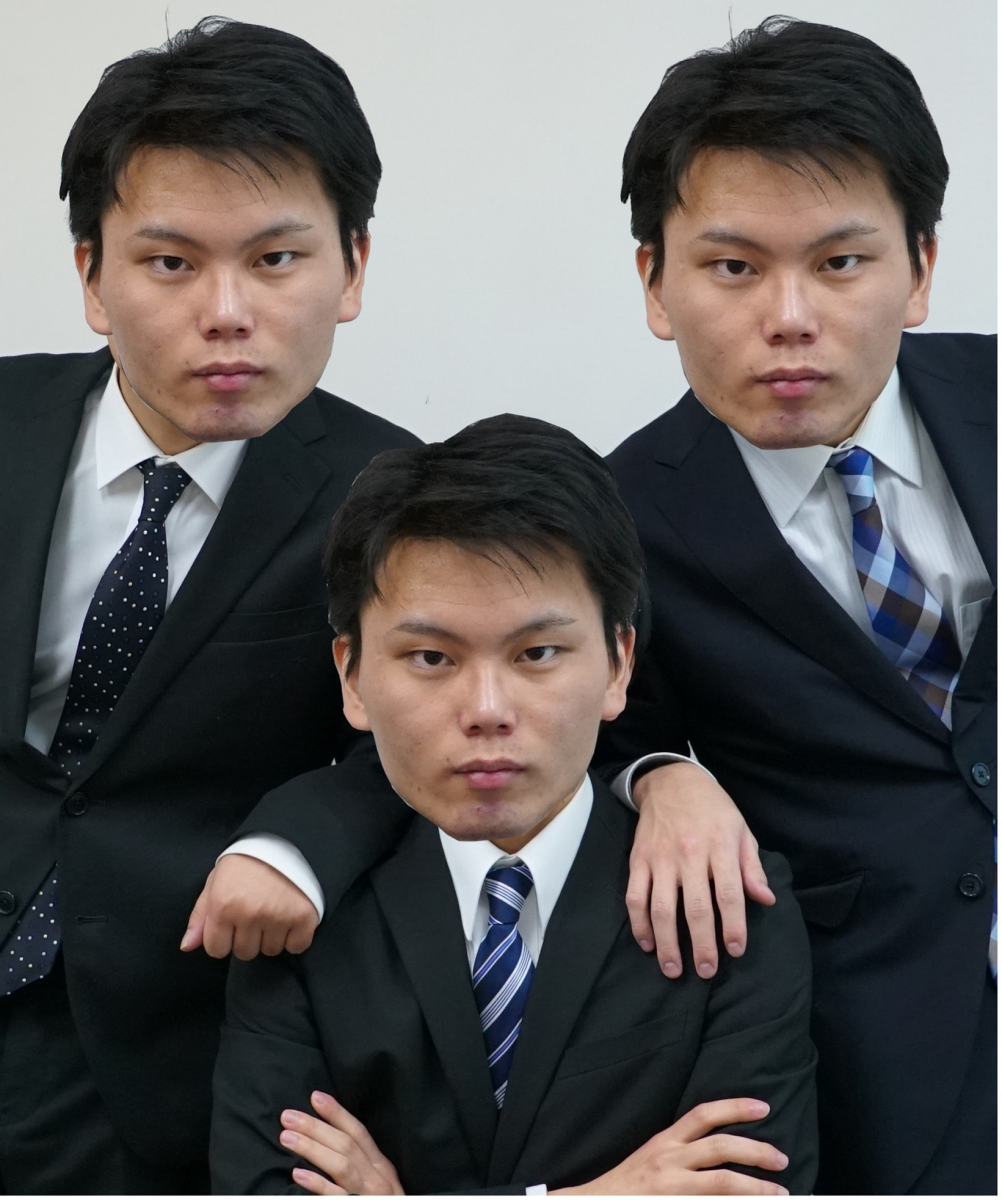
An example of clone faces. This is the first author’s face.

If it becomes easy to overcome the uncanny valley, what will the future be like? A major goal in the creation of human-like robots is to introduce robots into human society by making it easier for people to interact with them. In the future, it may be normal for humanoid robots that are indistinguishable from humans to be present in society. To spread humanoid robots quickly and widely, human-like androids must be mass produced. Thus far, Pepper has been mass-produced and introduced into offices, commercial facilities, convenience stores, and schools [9]. If the mass production of humanoid robots that look exactly the same as human beings is realized, humanoid robots that have faces with the exact same appearance may be present in the same location (Fig 1). This issue is not limited to robots, but could also be caused by the development of life sciences. When human cloning becomes technologically possible, we may find ourselves in a situation in which people with the exact same faces exist in society. In fact, the cloning of macaque monkeys, which is a species close to humans, has been successful [10], although the application of cloning technology to human beings is currently ethically prohibited. Hence, it would not be surprising to be confronted with situations in which people or human-like products have faces with the exact same appearance in the future (here, we call these clone faces); in such a future, what impression will we feel from these clone faces? Given the impact of the development of technology on human beings like the uncanny valley, predicting possible phenomena and our psychological responses to them in the future is important to create a society in which technologies and human beings can coexist before such new technologies are introduced into society. Therefore, investigating the psychological reactions elicited by clone faces would be beneficial for the smooth introduction and presence of such technologies.

Most previous studies about facial impressions used a single face for stimuli [11, 12], though some researched have used multiple faces for stimuli and presented them simultaneously. These studies found that presenting multiple faces influenced facial attractiveness. For example, female individuals are evaluated as more attractive in a group than when they are presented individually [13]. Moreover, observers rated overall group attractiveness higher than the average attractiveness of group members [14]. These studies used multiple faces of different individuals (i.e., a group) and mainly investigated the impressions of individuals in the group. Therefore, it remains unclear what impressions clone faces elicit.

Previous findings concerning the uncanny valley [3] might be helpful in the consideration of the impressions elicited by clone faces. As stated above, the uncanny valley is the phenomenon through which humanoids elicit unpleasant and negative emotions in viewers when their appearance becomes similar to that of humans to some extent. Interestingly, the uncanny valley can also be found in other strange and ambiguous objects such as computer graphics and food [5, 15]. Two main mechanisms of the uncanny valley have been proposed: the inconsistency of realism [16] and categorization-based stranger avoidance [17, 18]. MacDorman and Chattopadhyay [16] found that inconsistency in the realism of facial features (e.g., real eyes with artificial skin) gave eerier and colder impressions and hence play key roles in the uncanny valley phenomenon. However, Yamada et al. [18] found that objects that are difficult to categorize elicit eeriness, indicating that categorical difficulty contributes to the uncanny valley. Although these two theories remain under debate, they provide important suggestions for predicting impressions regarding clone faces. When we encounter clone faces in daily situations, we may avoid them due to the stranger phenomenon because they have inconsistent realism or are difficult to categorize into a well-known class. As a result, negative emotions may arise when observing clone faces.

Clone faces may elicit positive as well as negative emotions due to processing fluency. An object the information of which is easily and fluently processed evokes positive reactions (see [19], for a review). The mere exposure effect [20] is one of the examples of the positive reaction stemming from processing fluency [21]: repeated exposure to a stimulus increases perceptual fluency and as a result, liking for the stimuli increases. These findings indicate that clone faces might also be processed more fluently because when observing clone faces, we repeatedly process only one type of face. However, when observing different faces, we must process each face individually. Therefore, clone faces might be more easily processed than different faces. As a result, clone faces would be evaluated more positively than different faces.

Study 1 investigated the impressions clone faces induce. We defined two hypotheses regarding the impression of clone faces: the first assumes that they may be seen as improbable and potential threats and hence would be negatively evaluated [16–18]. The other hypothesis asserts that clone faces would be positively evaluated because they are fluently processed [21]. We tested these hypotheses by comparing the perceived eeriness of six individuals with clone faces to that of a single person and six individuals with different faces with each other. In Studies 2–5, we further investigated what influences the impression of clone faces from the perspective of realism by manipulating the number of clone faces, species, identity, and the contextual scene. Finally, we investigated personality traits involved with the impression of clone faces in Study 6.

## Study 1

### Method

#### Participants

121 Japanese people were recruited via Yahoo! Crowdsourcing and participated in the experiment online. The purpose of the study was not revealed to the participants. The experiment was conducted according to the principles expressed in the Helsinki Declaration. The ethics committees of Kyushu University approved the study protocol (approval number: 2013–008).

#### Stimulus and procedure

Three kinds of stimuli were developed: images with six people with clone faces (clone image), six people with different faces from each other (non-clone image), and one person (single image). First, we obtained contextual images from Google searches with a single person (6 images) or six persons (7 images). One of the six-people images was used for the non-clone image and the others were used for the clone images. Then, we selected six face images of Japanese, white, and black people from a database and trimmed the area of the face including the hair around the facial contour. Finally, the trimmed face images were pasted on bodies in the contextual images. We used Adobe Photoshop CS5 for photo editing. We created clone and single images using all of the faces, producing 18 clone and single images. Moreover, we created non-clone images for each race; hence, three non-clone images existed in total. Note that we not only edited the clone images but also non-clone images so that the visual noise produced in the editing of the image was almost equivalent between the clone and non-clone images. All the people in the images were men because editing and controlling female faces was difficult owing to various features (e.g., hair). These stimuli were presented on a computer screen. We can provide images used in this and subsequent experiments of the present study upon request.

Participants were asked to evaluate the subjective eeriness (1: not at all eerie, 7: very eerie), emotional valence (1: very unpleasant, 7: very pleasant), and realism (1: not at all real, 7: very real) of the images on a 7-point Likert-scale. We set easy calculations (e.g., 73–44 =?) as attention check questions (ACQs) to detect satisfiers [22]. The order of presentation of each image and ACQ was randomized across the participants.

#### Data analysis

We excluded participants who gave incorrect answers to one or more ACQs. We computed the average of the subjective eeriness, emotional valence, and realism scores for each condition. We conducted one-way analyses of variance (ANOVA) on each score with the image type as a within-factor and calculated η_*p*_^2^s. If the main effect was significant, we conducted multiple comparisons using Shaffer’s modified sequentially rejective Bonferroni methods and calculated Cohen’s *dz*. The alpha level was .05.

### Results and discussion

**Fig 2:**
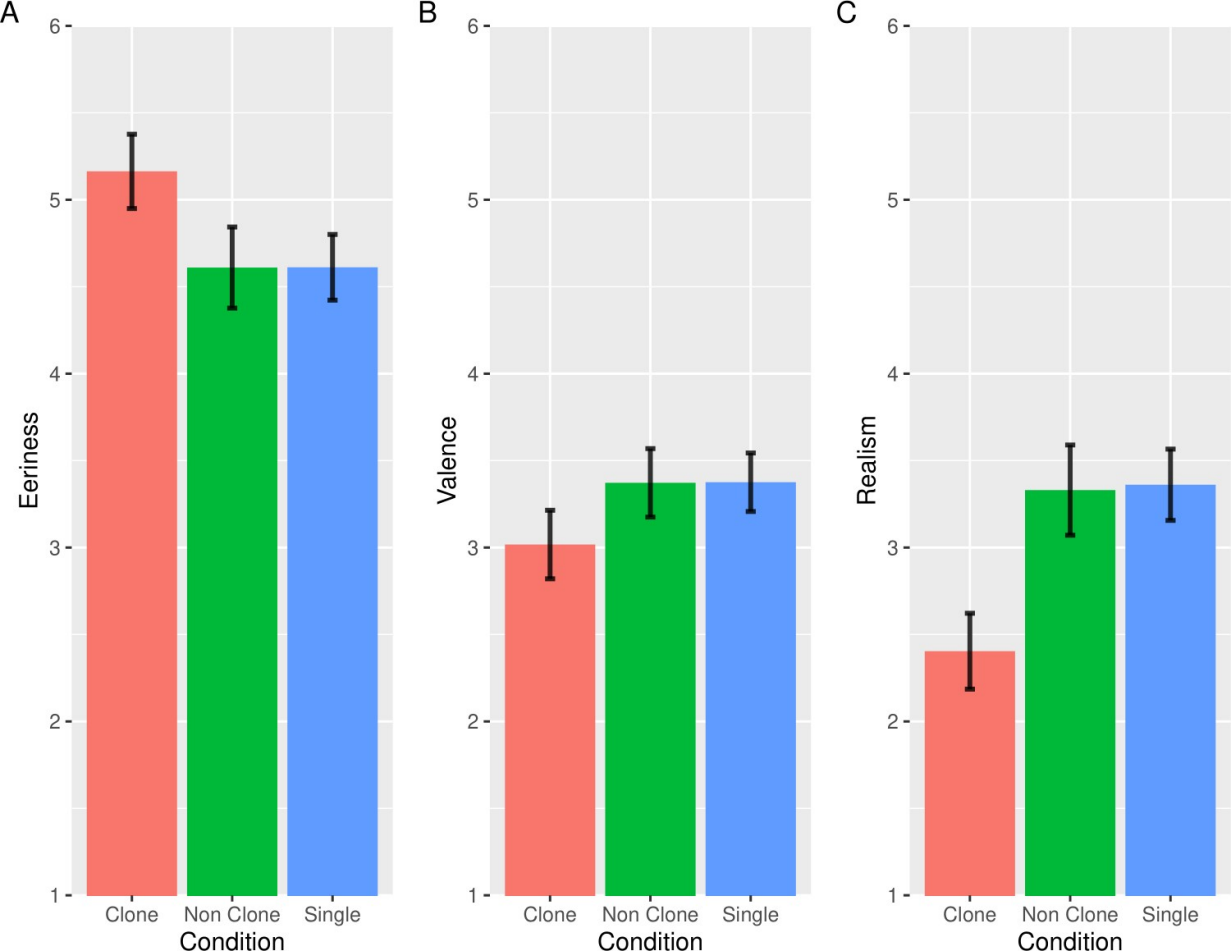
The results of the eeriness, valence, and realism evaluation in Study 1. Error bars indicate the standard errors of the mean. The vertical axes indicate the mean eeriness (A), valence (B), realism scores (C) for images in each condition. The lower scores indicate more negative and improbable evaluation.

We excluded 8 participants according to the criteria indicated in *Data Analysis* and hence we used the data of 112 participants for the statistical analysis (54 women; mean age 26.75 years). Fig 2 shows the mean of the eeriness, valence, and realism scores. The results of ANOVA on the subjective eeriness scores revealed that the main effect of image conditions was significant (*F*(2, 222) = 33.76, *p* < .001, η_*p*_^2^ = .23). Multiple comparisons revealed that the eeriness of the clone condition was significantly higher than the non-clone condition and single condition (clone vs. non-clone: *t*(111) = 6.29, *p* < .001, Cohen’s *dz* = 0.59; clone vs. single: *t*(111) = 7.46, *p* < .001, Cohen’s *dz* = 0.70). However, there was no significant difference between the non-clone and single conditions (*t*(111) = 0.21, *p* = .98, Cohen’s *dz* = 0.002).

The results of ANOVA on the emotional valence scores revealed that the main effect of image conditions was significant (*F*(2, 222) = 24.90, *p* < .001, η_*p*_^2^ = .18). Multiple comparisons revealed that the clone condition was significantly more negative than the non-clone condition and single condition (clone vs. non-clone: *t*(111) = 5.48, *p* < .001, Cohen’s *dz* = 0.52; clone vs. single: *t*(111) = 6.07, *p* < .001, Cohen’s *dz* = 0.57). However, there was no significant difference between the non-clone and single conditions (*t*(111) = 0.07, *p* = .95, Cohen’s *dz* = 0.01).

The results of ANOVA on the realism scores revealed that the main effect of image conditions was significant (*F*(2, 222) = 54.79, *p* < .001, η_*p*_^2^ = .33). Multiple comparisons revealed that the realism of the clone condition was significantly lower than the non-clone condition and single condition (clone vs. non-clone: *t*(111) = 7.61, *p* < .001, Cohen’s *dz* = 0.72; clone vs. single: *t*(111) = 9.19, *p* < .001, Cohen’s *dz* = 0.87). However, there was no significant difference between the non-clone and single conditions (*t*(111) = 0.37, *p* = .71, Cohen’s *dz* = 0.04).

These results indicated that the clone images were perceived as eerie, improbable, and negative compared to the other images. Based on these results, we rejected the possibility that the clone images would be evaluated positively because of high processing fluency [21]. We identified a new phenomenon through which clone faces induce negative impressions. We called this phenomenon the *clone devaluation effect*. As discussed in the Introduction section, the clone devaluation effect may occur because clone faces are strange and we try to avoid them to protect ourselves (stranger avoidance mechanism; [15, 17, 18, 23, 24]).

In regard to realism, the results showed that clone faces were significantly lower than non-clone faces and single faces, which suggests that participants considered the images of clone faces as improbable scenery compared to those of non-clone faces and single faces. In addition, the results of Study 1 suggested that the improbability of clone faces was related to their eeriness because the tendency of realism scores was consistent with that of eeriness scores across conditions. The improbability of clone faces may reflect their improbability in a highly realistic context like photographs. Indeed, previous studies on the uncanny valley have found that the inconsistency of realism in objects elicits feelings of eeriness [16]. Thus, it is possible that the clone devaluation effect is caused by the difference in reality between the context and clone faces.

Moreover, to investigate whether the clone devaluation effect occurs in not only Japanese but also other ethnic groups, we conducted a supplementary experiment with white people as participants using the same method as in Study 1 (see S1 Text for details). The results obtained were similar to Study 1, which suggests that the clone devaluation effect also emerged among participants of other ethnicities. Therefore, it is possible that the clone devaluation effect has robustness across ethnic groups to some extent. However, we note that this evidence should be judged carefully because the sample size of the supplementary experiment was small (*N* = 35).

One may argue that the clone devaluation effect stems from visual noise due to image editing. If the clone devaluation effect occurred for this reason, the subjective eeriness and unpleasantness of the images would be comparable with that of non-clone images because these two kinds of the images were edited in the same manner. However, the results of Study 1 revealed that a significant difference was found not only between the clone and single images but also between the clone and non-clone images. In addition, there was no significant difference between the non-single and single images. Hence, visual noises due to editing cannot explain the clone devaluation effect.

Study 1 showed that clone faces induce negative impressions when only six people were in in the image. Generally, as the number of the clone faces increases, the scenes should be seen as increasingly strange. Therefore, it is naturally predicted that an increase of the clone faces would enhance the clone devaluation effect. Study 2 addressed this issue; we manipulated the number of people by two, three, four, and five people in the clone and non-clone images and compared the impressions between them.

## Study 2

### Method

#### Participants, stimulus, procedure, and data analysis

179 Japanese people were recruited via Yahoo! Crowdsourcing and participated in the experiment online. Stimuli were created in almost the same manner as in Study 1. In Study 2, we only used Japanese faces and did not use single images. We obtained contextual images with two, three, four, or five persons (12 images each) from a Google search and pasted Japanese faces used in Study 1 on the bodies of the people in the contextual images. The stimuli images consisted of two kinds of clone faces (clone and non-clone) and four groups of persons (two, three, four, and five people). Half of the contextual images were used for the clone condition and the remaining six images were used for the non-clone condition; in total, we presented 48 images. The procedure and data analysis were identical to those used in Study 1 except that the emotional valence item was removed. This is because we considered the emotional valence item as unnecessary in Study 2, considering that we could confirm that clone faces are evaluated negatively in Study 1 using the emotional valence item. We performed two-way repeated-measures ANOVAs on the subjective eeriness and realism scores with clone faces and the number of people as within- factors to test the influence of the number of people on the clone devaluation effect.

### Results and discussion

**Fig 3:**
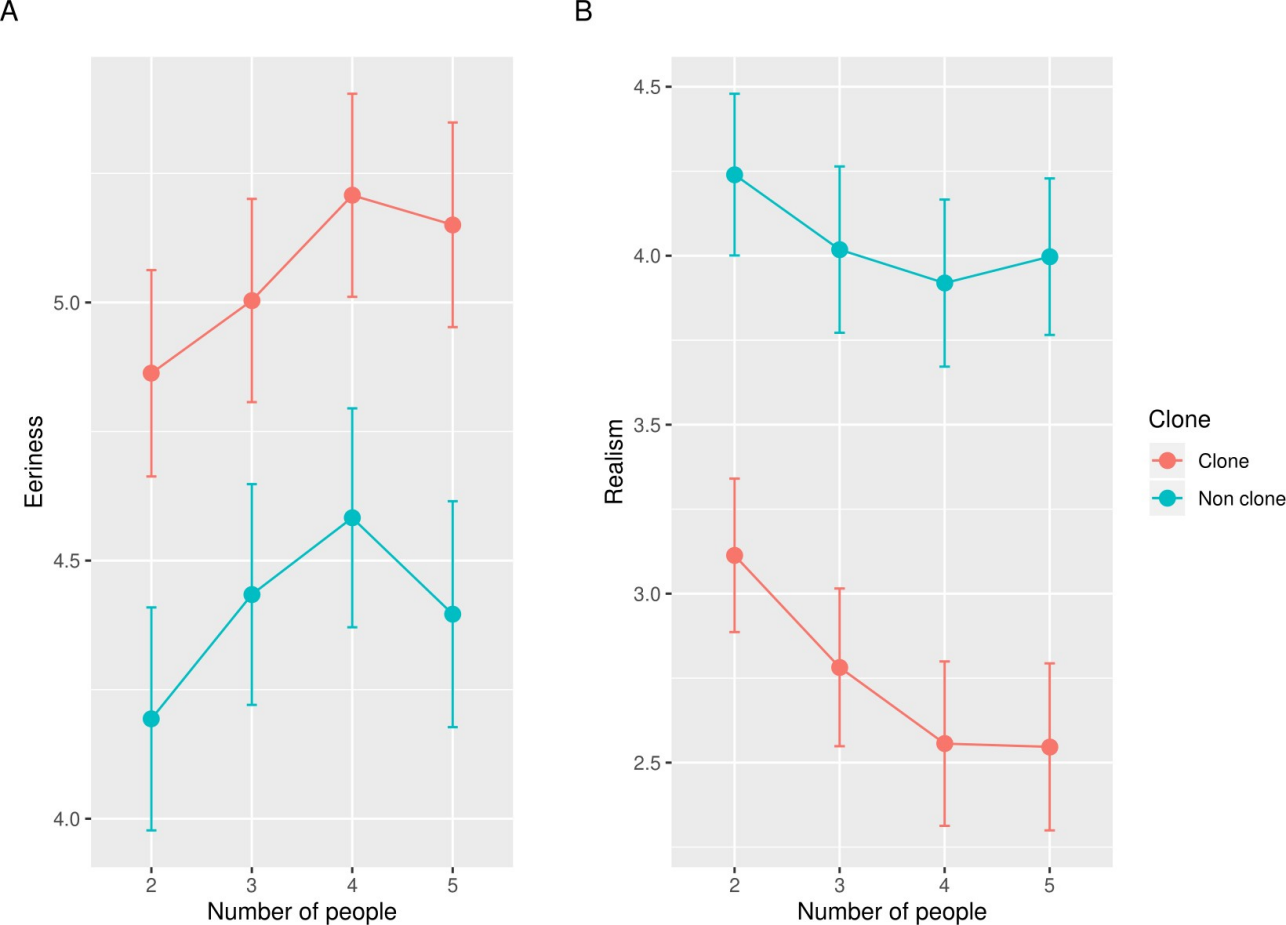
The results of the eeriness and realism evaluation in Study 2. Error bars indicate the standard errors of the mean. The vertical axes indicate the mean eeriness (A), the realism scores (B) for images in each condition. The lower scores in realism indicate more improbable evaluation.

We excluded 4 participants who gave incorrect answers to one or more ACQs and hence we used the data of 175 participants for the statistical analysis (92 women; mean age 39.26 years). Fig 3 shows the mean of the eeriness and realism scores. The results of the ANOVA on the eeriness scores revealed that the main effect of clone faces, *F*(1, 174) = 61.12, *p* < .001, η_*p*_^2^ = .26, the main effect of the number of people, *F*(3, 522) = 40.54, *p* < .001, η_*p*_^2^ = .19, and the interaction *F*(3, 522) = 2.90, *p* < .05, η_*p*_^2^ = .02, were significant. The significant main effect of clone faces indicates that the clone images were eerier than non-clone images. The simple main effect of the number of people was significant in the clone images (*F*(3, 522) = 18.84, *p* < .001, η_*p*_^2^ = .10). Multiple comparisons revealed that the eeriness of clone images with four and five people was higher than those with two and three people (four vs. two: *t*(174) = 5.64, *p* < .001, Cohen’s *dz* = 0.43; four vs. three: *t*(174) = 4.95, *p* < .001, Cohen’s *dz* = 0.37; five vs. two: *t*(174) = 4.86, *p* < .001, Cohen’s *dz* = 0.37; five vs. three: *t*(174) = 3.37, *p* < .001, Cohen’s *dz* = 0.25) and the eeriness of clone images with three people was higher than those with two people (*t*(174) = 5.64, *p* < .001, Cohen’s *dz* = 0.20). On the other hand, there was no significant difference in the subjective eeriness of the clone images between four and five people (*t*(174) = 1.43, *p* = .16, Cohen’s *dz* = 0.11). The simple main effect of the number of people was also significant in the non-clone images (*F*(3, 522) = 18.84, *p* < .001, η_*p*_^2^ = .14). Multiple comparisons showed that the eeriness of non-clone images with four was significantly higher than those of any other image (four vs. two: *t*(174) = 8.65, *p* < .001, Cohen’s *dz* = 0.65; four vs. three: *t*(174) = 4.95, *p* < .001, Cohen’s *dz* = 0.35; four vs. five: *t*(174) = 4.73, *p* < .001, Cohen’s *dz* = 0.27). Moreover, the eeriness of non-clone images with three and five people was significantly higher than those with two people (three vs. two: *t*(174) = 4.73, *p* < .001, Cohen’s *dz* = 0.36; five vs. two: *t*(174) = 5.27, *p* < .001, Cohen’s *dz* = 0.40) while there was no significant difference in the subjective eeriness of the non-clone images between three and five people (*t*(174) = 0.89, *p* = .37, Cohen’s *dz* = 0.07).

The results of ANOVA on the realism scores revealed that the main effect of clone faces, *F*(1, 174) = 80.04, *p* < .001, η_*p*_^2^ = .32, the main effect of number of people conditions, *F*(3, 522) = 38.63, *p* < .001, η_*p*_^2^ = .18, and the interaction, *F*(3, 522) = 5.35, *p* < .01, η_*p*_^2^ = .03, were significant. The significant main effect of clone faces indicates that the non-clone images were more real than the clone images. The simple main effect of the number of people was significant in the clone images (*F*(3, 522) = 26.38, *p* < .001, η_*p*_^2^ = .13). Multiple comparisons revealed that the realism scores of clone images with four and five people were lower than with two and three people (four vs. two: *t*(174) = 6.15, *p* < .001, Cohen’s *dz* = 0.46; four vs. three: *t*(174) = 4.52, *p* < .001, Cohen’s *dz* = 0.34; five vs. two: *t*(174) = 5.50, *p* < .001, Cohen’s *dz* = 0.42; five vs. three: *t*(174) = 3.84, *p* < .001, Cohen’s *dz* = 0.29). However, there was no significant difference between four and five people on the realism of the clone images (*t*(174) = 0.25, *p* = .80, Cohen’s *dz* = 0.02). The simple main effect of the number of people was also significant in the non-clone images (*F*(3, 522) = 14.97, *p* < .001, η_*p*_^2^ = .08). Multiple comparisons showed that the realism of non-clone images with two was significantly higher than those of any other image (two vs. three: *t*(174) = 4.71, *p* < .001, Cohen’s *dz* = 0.36; two vs. four: *t*(174) = 6.24, *p* < .001, Cohen’s *dz* = 0.47; two vs. five: *t*(174) = 4.75, *p* < .001, Cohen’s *dz* = 0.36). There was also a significant difference between three and four people (*t*(174) = 2.26, *p* < .05, Cohen’s *dz* = 0.17). However, there was no significant difference between three and five people, *t*(174) = 0.37, *p* = .71, Cohen’s *dz* = 0.03, and four and five people, *t*(174) = 1.52, *p* = .13, Cohen’s *dz* = 0.11, on the realism of the non-clone images.

The results of Study 2 indicated that the clone images were eerier and more improbable than non-clone images in all of the conditions with different numbers of people. Thus, we replicated the results of Study 1. In addition, these results suggest that even two clone faces are enough to cause the clone devaluation effect.

In the clone images condition, the subjective eeriness was higher as the number of people with the clone faces increased, although there was no difference in the subjective eeriness between four and five people. This tendency was also found in the realism of people with the clone faces. These results suggest that the clone devaluation effect is saturated at four people. Moreover, in the clone images, the subjective eeriness and improbability increased as the number of people increased, whereas this tendency was not found in the non-clone images. Briefly, the subjective eeriness and realism covaried in the clone image, indicating that reality is important for the clone devaluation effect.

Studies 1 and 2 manipulated the duplication of faces and the number of clone faces, given that participants recognized the duplication of faces. We consistently showed that clone faces increased in improbability as well as eeriness. In the real world, people can adequately distinguish human faces and clone faces do not generally exist. Namely, the clone faces deviate from the regular state of the external world and most people have never encountered them; this improbability might be a key to the clone devaluation effect. Then, would the clone devaluation effect be induced by clone faces when they are originally indistinguishable? In this case, the clone faces should be natural in a sense. If the clone devaluation effect was caused by the improbability of the clone faces, the clone devaluation effect would not occur in the indistinguishable clone faces. A previous study showed that, even in dog experts (i.e. dog breeder and trainer), the recognition accuracy of dog faces was lower than that of human faces [25]. In other words, it is too difficult for humans to distinguish the faces of other species. Therefore, we would clarify the influence of distinguishableness of faces on the clone evaluation effect by using dog faces. If the improbability of the clone faces was key to the clone devaluation effect, the clone devaluation effect did not occur in dog faces; therefore, we predicted that the eeriness of the images with the dogs’ clone faces would be equivalent to those with different dogs’ faces.

## Study 3

### Method

#### Participants, stimulus, and procedure

200 Japanese people were recruited via Yahoo! Crowdsourcing and participated in the experiment online. We obtained two images in which five dogs existed from a Google search. One was used for the clone image and the other was used for the non-clone image. Each image consisted of the same breeds of dogs (Shiba Inu and Siberian husky). We edited only the clone image to duplicate an individual’s face; we trimmed one of the faces and imposed it over the other bodies.

The procedure was identical to that of Study 2. To confirm whether the subjective eeriness and realism scores differed between the clone and non-clone images, we conducted two-tails paired *t*-tests and reported Cohen’s *dz*. Moreover, it was highly possible that the scores were equivalent between the clone and non-clone images. To address this issue, we conducted equivalence tests (lower equivalence bounds = -0.5, upper equivalence bounds = 0.5, α = .05) when the *t*-tests found no significant differences. For the equivalence tests, we used the TOSTER package in R [26].

### Results and discussion

**Fig 4:**
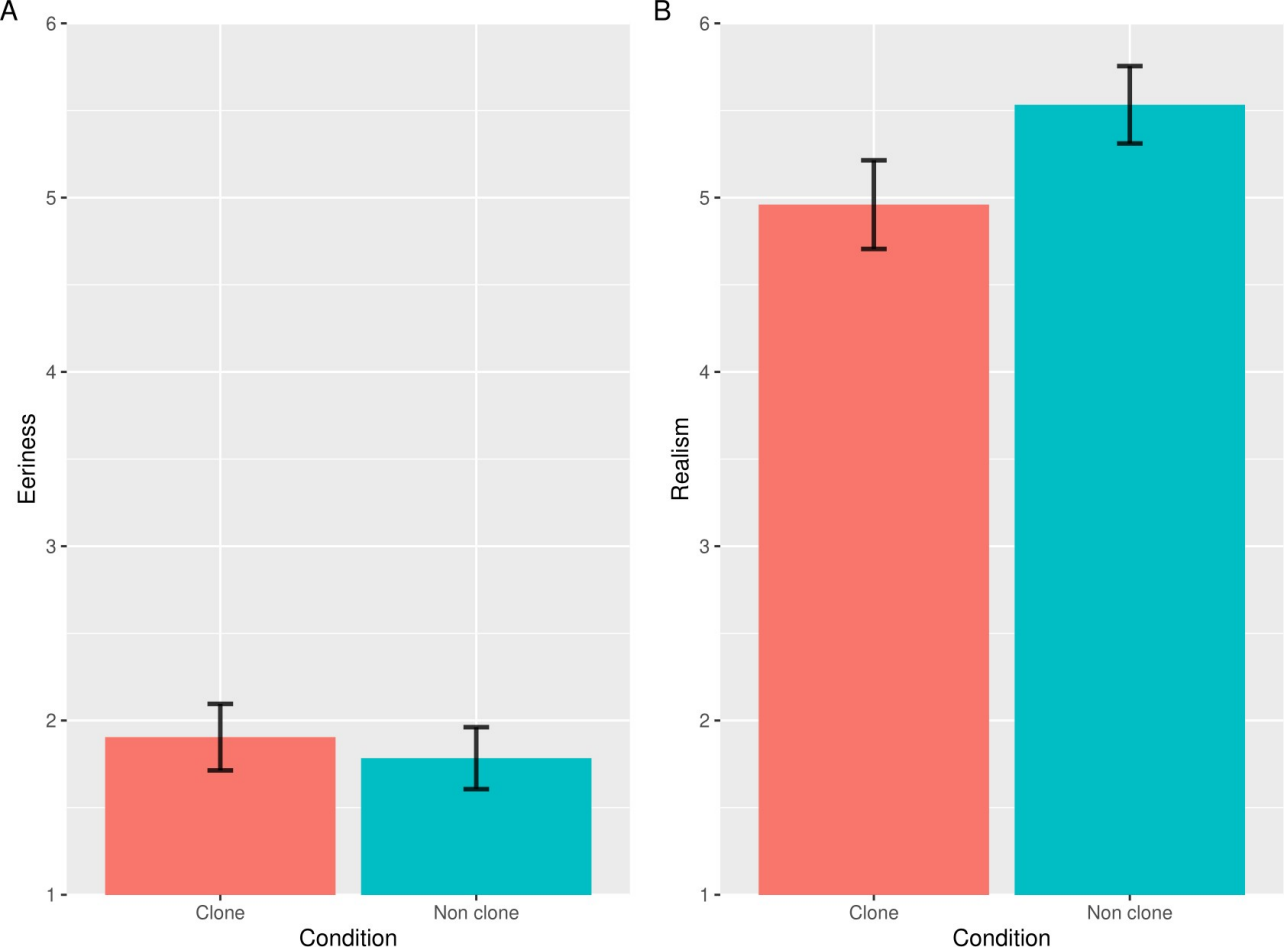
The results of the eeriness and realism evaluation in Study 3. Error bars indicate the standard errors of the mean. The vertical axes indicate the mean eeriness (A), the realism scores (B) for images in each condition. The lower scores in realism indicate more improbable evaluation.

We excluded one participant who gave incorrect answers to one or more ACQs and hence we used the data of 199 participants for the statistical analysis (64 women; mean age 43.83 years). Fig 4 shows the mean of the eeriness and realism scores. The results of the *t*-tests for the subjective eeriness scores showed that there was no significant difference between the clone and non-clone images (*t*(198) = 1.45, *p* < .15; Cohen’s *dz* = 0.10). Therefore, we conducted the equivalence tests and found that the eeriness of clone and non-clone images were significantly equivalent (*t*(198) = 5.61, *p* < 0.001). However, the results of the *t*-tests for the subjective realism scores showed that the clone image was significantly more improbable than the non-clone image (*t*(198) = 4.79, *p* < .001, Cohen’s *dz* = 0.34).

We have difficulties in distinguishing the individual faces of other species [25]. Considering this, the participants could not notice the differences in dog faces across individuals in the clone images because they innately could not distinguish dogs by their faces. As a result, the clone devaluation effect did not occur. These results suggest that facial discriminability is a key role in the clone devaluation effect.

However, there was a significant difference in realism between the clone and non-clone images. According to a previous study [25], the participants were less likely to notice the clone faces of dogs and thus, these results seem to be slightly odd. Perhaps the results were caused not by the clone faces but by other factors. For example, in Study 3, the head directions of all five dogs in the clone images were identical because of the editing of the image and the participants might consider this as strange. Importantly, subjective improbability stemming from recognizing the clone faces, not the other factors, led to the clone devaluation effect.

The results of Study 3 suggested that the clone faces were not always necessary to elicit the clone devaluation effect. Here, it was assumed that there were two possibilities. One is that the same facial features that clone faces have might induce the clone devaluation effect. The other possibility is that the duplication of identity between people with clone faces was important for the clone devaluation effect. To confirm this point, we used famous actresses and comedians of twin and non-twin Japanese people because twins share the almost same facial features with each other but do not have the same identity (i.e., the people in the images should be judged as different from each other). On the other hand, when non-twin people each have the same facial features, it should be perceived that their identity is duplicated (i.e., the people in the images should be judged as identical). If the duplication of the identity was important for the clone devaluation effect, the subjective eeriness of the duplicated twins’ faces would be lower than that of the duplicated non-twins’ faces. If the same facial features were important for the clone devaluation effect, the subjective eeriness would be equivalent between the twins’ faces and the duplicated non-twins’ faces.

## Study 4a

### Method

#### Participants, stimulus, and procedure

219 Japanese people were recruited via Yahoo! Crowdsourcing and participated in the experiment online. We obtained images in which two persons existed from Google search results. The image of Takuya and Kazuya in “The Touch,” who are a famous twin brother comic duo, and Kana Mikura and Mana Mikura, who are famous twin sister actors in Japan, were used for twin clone images. For the twin clone images, we imposed Takuya’s face and Mana’s face on Kazuya’s body and Kana’s body, respectively. We duplicated the faces of Fuminori Ujihara of “Rozan,” Yuki Iwai of “Haraichi,” Akira Ishida of “Non-Style,” who are members of each comic duo, and Haruka Ayase, who is a famous actress to create the non-twin clone images. For the non-twin images, we imposed their face on the body of the other person in each image.

Participants evaluated the subjective eeriness of the images and reported whether they knew the person in the images after evaluation. In addition to the ACQs, we excluded the data of the participants who did not know any of the persons in the images. To confirm whether the subjective eeriness differed between twin clone and non-twin clone images, we conducted two-tails paired *t*-tests and reported Cohen’s *dz*. Moreover, we conducted equivalence tests (lower equivalence bounds = -0.5, upper equivalence bounds = 0.5, α = .05) when the *t*-tests found no significant differences.

### Results and discussion

**Fig 5:**
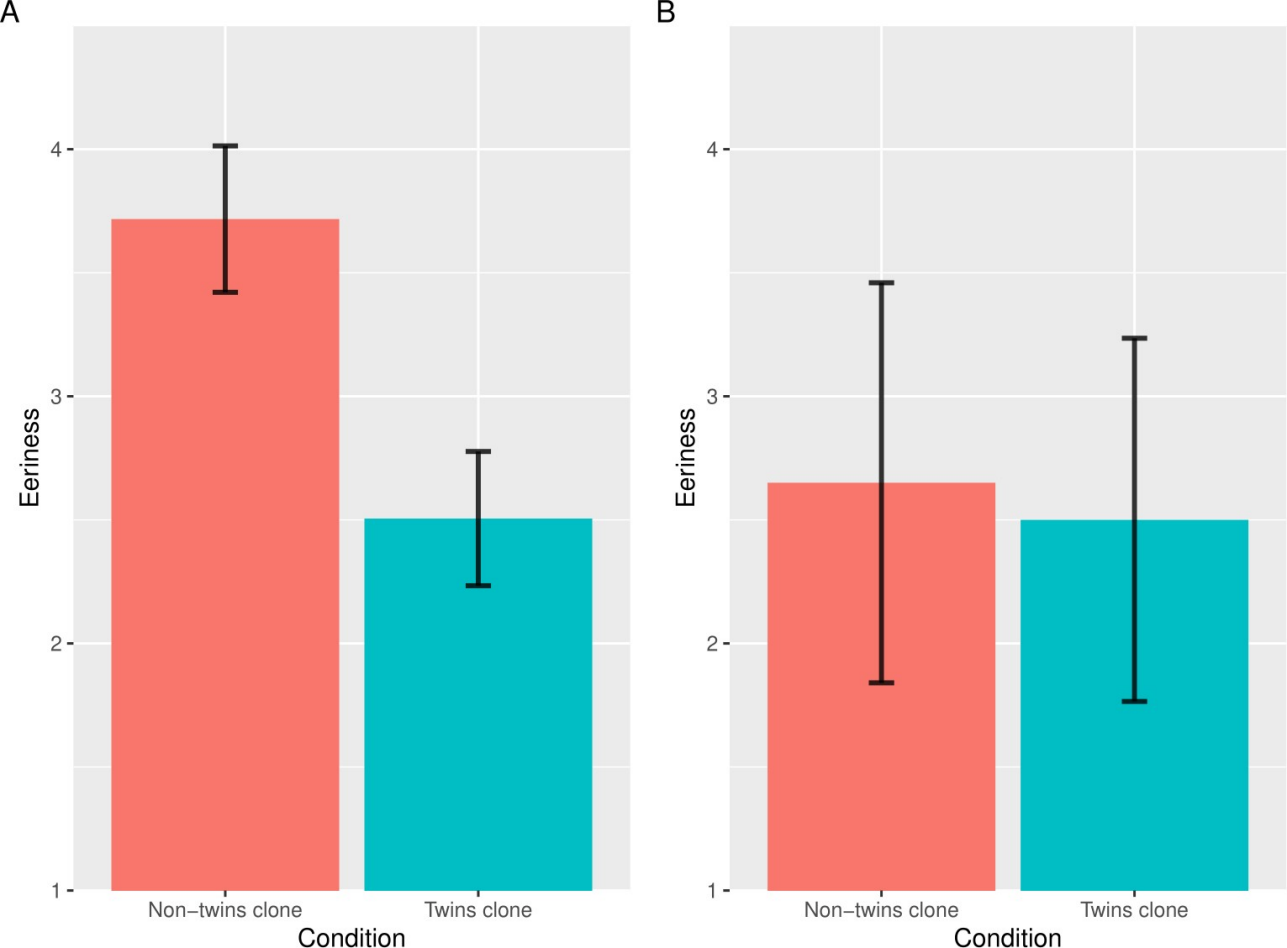
The results of the eeriness and realism evaluation in Study 4a. Error bars indicate the standard errors of the mean. Both of the vertical axes indicate the mean eeriness for images in each condition. The left graph was created from the data of the participants who knew all the people in the images (A). The right graph was created from the data of the participants who did not know any of the people in the images (B).

We excluded 120 participants according to the above criteria. Hence, we used the data of 99 participants for the statistical analysis (57 women; mean age 39.22 years). Fig 5A shows the mean of the eeriness score in the data of the participants who knew all the people in the images. The results of the paired *t*-tests showed that the eeriness of the twin clone condition was significantly lower than the non-twins clone condition (*t*(98) = 8.60, *p* < .001, Cohen’s *dz* = 0.86). Thus, even when the faces were duplicated, the participants judged their identities as different and their subjective eeriness decreased. Moreover, we post-hoc analyzed the data of the ten participants who did not know any person in the images. Fig 5B shows the mean of the eeriness score in the data of the participants who did not know any of the people in the images. The analysis showed that the difference between the twins clone condition and the non-twins clone condition were not significant (*t*(9) = 0.67, *p* = .52, Cohen’s *dz* = 0.21). Briefly, when the participants did not know the two persons in the twins-clone images as twins, they should be judged as having the same identity and hence the clone devaluation effect survived. Taken together, these results suggest that duplicated identities, rather than duplicated facial features, play key roles in the clone devaluation effect.

In Study 4a, we used widely known persons as stimuli and we found different responses between those who knew the certain persons as twins and those who did not; in those who did not know the certain persons as twins, the subjective eeriness of the twin clone image was high and was not significantly different from that of the non-twin clone image. Based on this, a new prediction was raised: when the participants were instructed that clone faces were multiplets, the subjective eeriness of the clone images would decrease. Thus, in Study 4b, we investigated whether the clone devaluation effect would be weakened by instruction and used the stimuli (i.e., not celebrities) from Study 2.

## Study 4b

### Method

#### Participants, stimulus, and procedure

We recruited participants via Yahoo! Crowdsourcing. Although we planned to recruit about 250 participants in each group, the sample size of the instruction group became too large because we overscheduled the recruitment. As a result, 692 people were assigned to the instruction group and 201 people were assigned to the non-instruction group; 893 Japanese people participated in total. The stimuli were identical to Study 2 that the participants in the instruction group were told that the clone faces in the images were multiplet from twin to quintuplet. To examine whether the effect of the instruction was found, we performed two-way mixed ANOVAs on the subjective eeriness and realism scores with the image type (clone *vs*. non-clone) as a within-participant factor and the group (instruction *vs*. non-instruction) as a between-participant factor.

### Results and discussion

**Fig 6:**
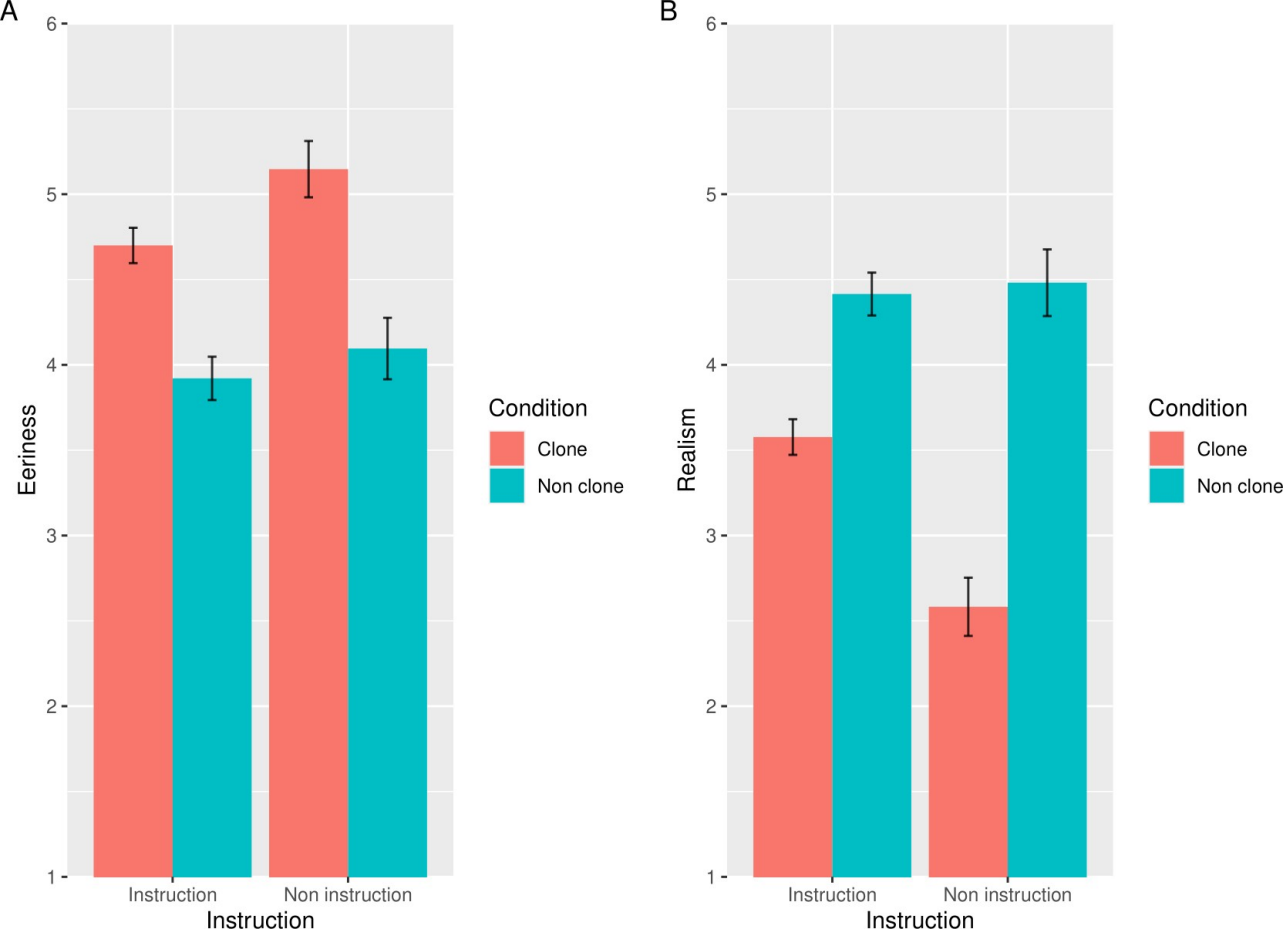
The results of the eeriness and realism evaluation in Study 4b. Error bars indicate the standard errors of the mean. The vertical axes indicate the mean eeriness (A), with realism scores (B) for images in each condition. The lower scores in realism indicate more improbable evaluation.

We excluded 12 participants who gave incorrect answers to one or more ACQs and hence we used the data of 881 participants for the statistical analysis (437 women; mean age 39.78 years); 630 people (346 women, mean age 38.21 years) in the instruction group and 251 people (91 women, mean age 43.70 years) in the non-instruction group. Fig 6 shows the mean of the eeriness and realism scores in the instruction and non-instruction groups. The results of the ANOVA on the subjective eeriness score showed the significant main effects of group, *F*(1, 879) = 10.70, *p* < .01, η_*p*_^2^ = .012, and image type, *F*(1, 879) = 303.53, *p* < .001, η_*p*_^2^ = .26. The interaction between the group and image type was also significant (*F*(1, 879) = 6.71, *p* < .01, η_*p*_^2^ = .008). Post-hoc tests revealed that the simple main effect of the group was significant in the clone images, *F*(1, 879) = 20.45, *p* < .001, η_*p*_^2^ = .02, indicating that the subjective eeriness of the clone images was lower in the instruction condition than in the non-instruction condition. On the other hand, the simple main effect of the group was not significant in the non-clone images (*F*(1, 879) = 2.21, *p* = .14, η_*p*_^2^ = .002). In addition, the main effect of the image type was significant in both of the groups (instruction group: *F*(1, 629) = 182.76, *p* < .001, η_*p*_^2^ = .23; non-instruction: *F*(1, 250) = 162.27, *p* < .001, η_*p*_^2^ = .39), indicating that the subjective eeriness of the clone images was consistently significantly higher than non-clone condition.

The results of the ANOVA on the subjective realism score showed the significant main effects of group, *F*(1, 879) = 30.55, *p* < .001, η_*p*_^2^ = .037, and image type, *F*(1, 879) = 369.42, *p* < .001, η_*p*_^2^ = .30. The interaction between group and image type was also significant (*F*(1, 879) = 55.50, *p* < .001, η_*p*_^2^ = .06). Post-hoc tests revealed that the simple main effect of the group was significant in the clone images (*F*(1, 879) = 98.23, *p* < .001, η_*p*_^2^ = .10), indicating that the subjective realism of the clone images was lower in the instruction condition than in the non-instruction condition. However, the simple main effect of the group was not significant in the non-clone images (*F*(1, 879) = 0.31, *p* = .58, η_*p*_^2^ = .0004). In addition, the main effect of the image type was significant in both of the groups (instruction group: *F*(1, 629) = 121.57, *p* < .001, η_*p*_^2^ = .16; non-instruction: *F*(1, 250) = 248.68, *p* < .001, η_*p*_^2^ = .50), indicating that the subjective realism of clone condition was consistently significantly lower than non-clone condition in both the instruction condition and the non-instruction condition.

The clone devaluation effect in the instruction group was weaker than in the non-instruction group. In addition, we consistently indicated the correspondence of the tendency of the eeriness with that of realism, as described above. The results of Study 4b suggest that the cancellation of same the identities attenuated the clone devaluation effect, supporting the idea that the same identities, rather than that of facial features, drives the clone devaluation effect.

Through Studies 1 to 4, we used photographic images as stimuli with clone faces existed in a highly realistic context of daily scenes. In other words, when the scene in which clone faces were present was close to the real world, people evaluated clone faces as improbable and eerie. This suggests that a highly realistic context increases the improbability of clone faces and in turn, the clone devaluation effect occurred. In line with the suggestion, even the clone faces would be less eerie if there are improbable objects (i.e., clone faces) in a low realistic context. Therefore, in Study 5, we used clone faces drawn in anime and cartoon as stimuli to investigate the influence of scene context to observe clone faces on the clone devaluation effect. The improbability of clone faces may have been decreased by anime and cartoon image; hence we predicted that clone faces drawn in anime and cartoons would be less eerie and improbable than that of those in photographic images.

## Study 5

### Method

#### Participants, stimulus, and procedure

208 Japanese people were recruited via Yahoo! Crowdsourcing and participated in the experiment online. We obtained Japanese animation and cartoon images (cartoon clone condition) in which three, four, or six characters existed, from a Google search. The anime characters had appeared in popular Japanese anime (i.e., “Touch”, “Osomatsu-kun”, and “Osomatsu-san”)^1^. The gender of all the characters was man. The characters in each image were indistinguishable. We selected picture images of photographic clone conditions in which three, four, or six characters existed, from stimuli used in Studies 1 and 2 so that the number of persons in the pictures corresponded with that of characters in the cartoon clone images. The procedure and data analysis were identical to those used in Study 2. To confirm whether the subjective eeriness and realism scores differed between the photographic clone and cartoon clone images, we conducted two-tails paired *t*-tests and reported Cohen’s *dz*. Moreover, we conducted equivalence tests (lower equivalence bounds = -0.5, upper equivalence bounds = 0.5, α = .05) when the *t*-tests found no significant differences.

### Results and discussion

**Fig 7:**
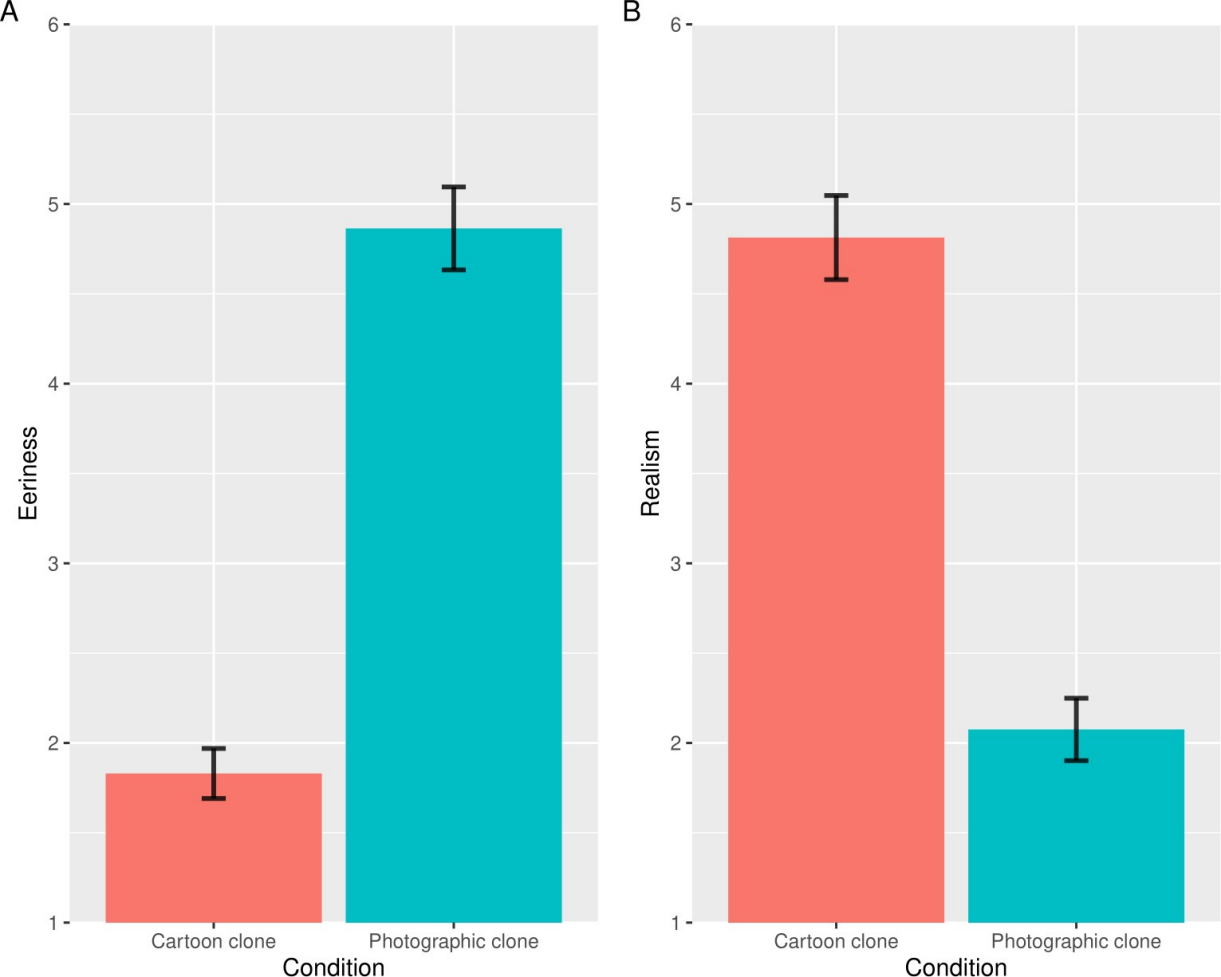
The results of the eeriness and realism evaluation in Study 5. Error bars indicate the standard errors of the mean. The vertical axes indicate the mean eeriness (A), with realism scores (B) for images in each condition. The lower scores in realism indicate more improbable evaluation.

We excluded 2 participants who gave incorrect answers to one or more ACQs and hence we used the data of 206 participants for the statistical analysis (65 women; mean age 43.57 years). Fig 7 shows the mean of the eeriness and realism scores in the cartoon and photographic conditions. The results of the paired *t*-test on the subjective eeriness score showed that the photographic clone condition was higher than the cartoon clone condition (*t*(205) = 25.95, *p* < .001, Cohen’s *dz* = 1.81). The results of the paired *t* test on the subjective realism score showed that the cartoon clone condition was lower than the photographic clone condition (*t*(205) = 17.68, *p* < .001, Cohen’s *dz* = 1.23). These results suggest that the probability of clone faces in an improbable context was higher than in realistic contexts and, as a result, the eeriness of clone faces drawn in cartoons decreased.

We have focused on the factors that modulate the clone devaluation effect in Studies 1 to 5. In this way, we consistently showed that the realism of clone faces corresponded to their eeriness. How does the improbability of clone faces affect their eeriness? One plausible explanation is that we tend to avoid improbable objects (i.e., clone faces) as strange, and eeriness occurs as one of the avoidance reactions. One previous study proposed that the uncanny valley is related to “stranger avoidance,” which is a tendency for people to avoid strangers who could potentially harm them [17, 18, 23]. This similar stranger avoidance system may apply to the clone devaluation effect; we may judge clone faces as improbable and stranger, and in turn, this may trigger an avoidance reaction. As a result, eeriness may be elicited. If this hypothesis is true, the eeriness of the clone faces would be related to the disgust reaction (i.e., one of the avoidance reactions to threat) as the similar fashion of the uncanny valley. A previous study on uncanny valley suggested that the feeling of the eeriness of stranger objects (i.e., humanoid) is related to disgust reaction on death [4]. Additionally, based on our hypothesis, the evaluation of realism may trigger disgust reactions. Therefore, the eeriness of clone face stemming from improbability would be related to disgust reaction.

Individual differences in disgust responses are called “disgust sensitivity.” The disgust sensitivity reflects the intensity of the disgust experience in exposure to the disgust elicitor and how negatively individuals evaluate the experience of disgust [27]. Here, we investigated the relationship between disgust sensitivity and the clone devaluation effect in Study 6. We predicted that the higher someone’s disgust sensitivity, the stronger the eeriness they would feel in the clone faces if the clone devaluation effect was related to disgust.

Moreover, we investigated what kinds of disgust sensitivity were related to the clone devaluation effect. There are mainly three types of disgust sensitivity depending on kinds of disgust elicitor: core disgust, animal-reminder disgust, and contamination disgust. Core disgust refers to the disgust response to primitive disgust elicitors such as food, animals, and excreta. Animal-reminder disgust refers to disgust response to disgust elicitors, such as death, physical damage, and sexual behavior, which remind humans of their animal traits. Contamination disgust refers to a disgust response to pollution, such as sanitary conditions and infections. Here, we could conduct an exploratory investigation on which disgust sensitivities to these disgust elicitors are related to the clone devaluation effect. This could indirectly reveal why the clone faces are eerie.

## Study 6

### Method

#### Participants, stimulus, and procedure

315 Japanese people were recruited via Yahoo! Crowdsourcing and participated in the experiment online. The stimuli were identical to those used in Study 2. The participants evaluated the subjective eeriness and realism of the images. After the evaluation, the participants were asked to answer the Japanese version of the Disgust Scale-Revised (DS-R-J: [28]) on a five-point Likert scale. The DS-R-J consists of 18 items used to measure disgust sensitivity based on the Disgust Scale-Revised developed by Haidt, McCauley, and Rozin [29]. This scale has a three-factor structure; core disgust, animal-reminder disgust, and physical and mental contamination disgust. To confirm which factors influence the clone devaluation effect, we conducted a multiple regression analysis (stepwise method) with the three factors as predictors of the eeriness of clone faces. In addition, in order to confirm how disgust sensitivity influences the clone devaluation effect, we conducted a mediation analysis with the realism of clone faces as a mediator.

### Results and discussion

We excluded 7 participants who gave incorrect answers to one or more ACQs and hence we used the data of 308 participants for the statistical analysis (127 women; mean age 41.83 years). Firstly, to confirm whether the clone devaluation effect occurred, a paired *t* test between clone images and non-clone images on the eeriness scores was performed, which revealed that the eeriness of clone images is significantly higher than that of non-clone images (*t*(307) = 13.13, *p* < .001, Cohen’s *dz* = 0.75). Following this, the realism of clone images is significantly lower than that of non-clone images (*t*(205) = 14.72, *p* < .001, Cohen’s *dz* = 0.84). Therefore, we could replicate the clone devaluation effect.

**Fig 8:**
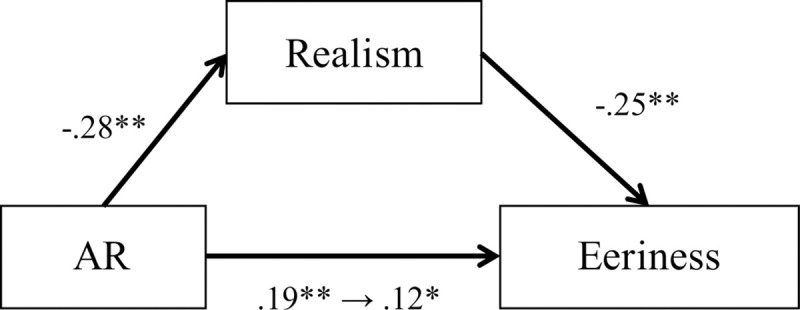
The results of the mediation analysis in Study 6. AR refers to animal-reminder disgust sensitivity. The numerical values around the arrows indicate standardized partial regression coefficients. An indirect effect is significant. *p < .05, *p < .01.

A multiple regression analysis (stepwise method) was performed with the three factors of the DS-R-J as predictors of the eeriness of clone faces. This regression showed that only animal-reminder disgust remained in the model (adjusted *R*^2^ = .03, *p* < .001) and a higher level of animal-reminder disgust sensitivity significantly predicted a higher eeriness of clone faces (β = .19, *p* < .001). In addition, we conducted a mediation analysis to test whether the realism of clone faces mediated the impact of the sensitivity to animal-reminder disgust on the eeriness of clone faces (Fig 8). We found significant links between all variables in the predicted directions. The sensitivity to animal-reminder disgust was positively associated with the eeriness of clone faces without the mediator (β = .19, *SE* = .08, *p* < .001). Additionally, the realism of clone faces was evaluated lower as the level of animal-reminder disgust sensitivity is higher (β = -.28, *SE* = .08, *p* < .001). The realism of clone faces had a significant impact on the eeriness of clone faces (β = -.25, *SE* = .04, *p* < .001). When the mediator was included in the regression, the impact of the sensitivity to animal-reminder disgust on the eeriness of clone faces was significantly reduced, but its impact reminded significant, which indicated that the indirect effect was significant and the eeriness of clone faces have a partial mediation effect (β = .12, *SE* = .09, *p* < .05; Sobel test: *z* = 3.33, *p* < .001).

Again, we have replicated the clone devaluation effect. We also found that only the animal-reminder disgust sensitivity in the three-factor structure of the DS-R-J positively predicted the evaluation on the eeriness of the clone faces. This indicates that the higher animal-reminder disgust sensitivity an individual has, the stronger the clone devaluation effect. Moreover, the influence of animal-reminder disgust sensitivity on the eeriness of the clone faces was mediated by the realism of the clone faces. Therefore, individuals who are sensitive to animal-reminder disgust tend to feel that the clone faces are eerie, which is due to the improbability of clone faces.

## General discussion

Our six studies investigated the impressions formed by clone faces and factors to influence the impression of clone faces. The participants evaluated six individuals with clone faces as eerier and more improbable than those with different faces and a person with a single face (Study 1). We called this negatively emotional response to clone faces the clone devaluation effect. This effect was stronger as the number of clone faces increased from two to four persons (Study 2). Moreover, this effect did not occur when each clone face was indistinguishable like animal faces (Study 3). It was also shown that the duplication of identity rather than facial features has an important role in this effect and clone faces with the duplication of identity were eerier (Studies 4a & 4b). The clone devaluation effect became weaker when clone faces existed in the lower reality of the context (Study 5). Furthermore, the eeriness of clone faces stemming from improbability would be positively predicted by disgust, in particular animal-reminder disgust (Study 6). Taken together, these results suggest that clone faces induce eeriness and that the clone devaluation effect is related to the realism and disgust reaction. We discuss the details of an assumed mechanism below.

Our findings provide evidence for the internal mechanisms of the clone devaluation effect. The results of Study 6 imply a relationship among realism, the eeriness of clone faces, and animal-reminder disgust sensitivity. The domain of animal-reminder disgust sensitivity includes the lack of the ideal appearance in a human body such as death or a damaged exterior form [30]. In addition to this idea, a previous study indicated that animal-reminder disgust is also involved in the eeriness of humanlike objects such as androids [31], the appearance of which is often improbable and strange. Objects with clone faces, even though they have an absolute appearance in human bodies, might be judged as strange because the clone faces are improbable. As a result, the eeriness is elicited in order to avoid any harm from such improbable and strange objects (i.e., clone faces). Taken together, the improbability of the clone faces is assumed to trigger a defensive reaction stemming from animal-reminder disgust, which in turn plays key roles in emotional reactions (i.e., eeriness).

Considering that improbability and strangeness are the keys to the phenomenon of the clone devaluation effect, a lack of humanity is another of the important parts of the effect. What is involved in the lack of humanity? The results of Studies 4a and 4b indicate that it is likely that this lack of humanity stems from duplication of identity, not facial features. Faces are important information for identifying individuals because human beings have a one-to-one correspondence between face and identity in principle. However, clone faces violate this principle, which may make us misjudge that the identity of people with clone faces should be the same. Thus, the duplication of identity is contrary to the principle of human beings and thus could be considered as a lack of humanity. In most of the present studies except for 4a and 4b, the clone devaluation occurred without the manipulation of identity; observers deduced duplication of identity from clone faces and, as a result, eeriness was elicited. However, we showed that the eeriness of the clone faces was low when clone faces were judged as multiplets; they have the same facial features. In this case, the participants were able to assign clone faces to different identities and thus eeriness was not evoked. Considering this, faces are the most critical cue of personal identity when there is no other special cue (e.g., details of siblings). This idea is consistent with the findings of previous studies [32, 33]. Taken together, the lack of humanity in the clone devaluation effect may stem from the duplication of identities inferred by clone faces.

**Fig 9:**
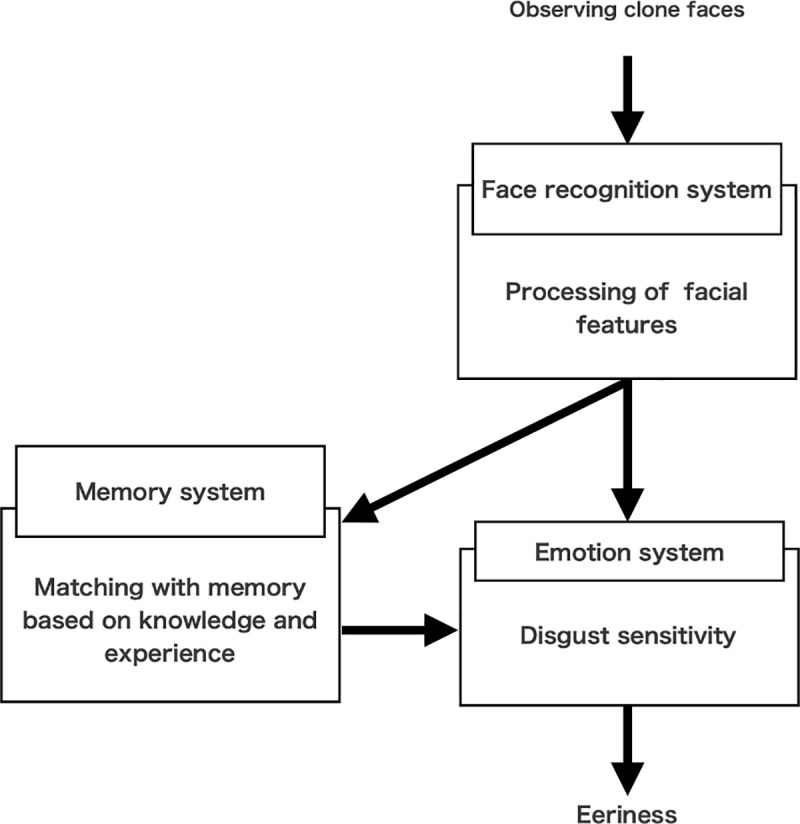
The mechanism of the clone devaluation effect proposed in the present study.

Considering the findings of our six studies, it was possible to some extent to speculate on a mechanism of the clone devaluation effect (Fig 9). Initially, when one observes clone faces, they are analyzed based on facial features. In this case, because they have identical facial features, they are judged as the same faces. This point is supported by Study 3, which showed that the clone devaluation effect did not occur when clone faces could not be analyzed and distinguished. Several models of aesthetic and facial processing argued that visual features were analyzed and abstracted from objects in the first stage [34–36]. Therefore, it is appropriate to assume that facial features are analyzed as the first stage of the facial processing system. After the judgment of clone faces, their information is processed in the memory system. In this stage, the realism of encountered clone faces is evaluated based on prior knowledge and experience. The results of studies 4a and 4b reflected this processing because the eeriness of famous or little-known multiplets’ faces was diminished even if they have clone faces. This stage is related to the reality of the background scene in which the clone faces exist, which supported the results of study 5’s that the eeriness and improbability of the photographic clone faces was higher than the cartoon clone faces. Although the present study only used the cartoon scene as less realistic scene than the photographic scene, we can investigate the influence of the background scene in further detail if the virtual reality space in which 3D avatars with clone faces existed was used as a more realistic scene. Finally, the improbability of clone faces induces avoidance reaction to strangers, which stems from disgust. Through this processing, unpleasant emotions (i.e., eeriness) toward clone faces are finally evoked.

The emergence of objects with clone faces is not a mere fantasy; they can be expected to appear in the near future. For example, when humanoid technology has further developed, the appearance of humanoids will be the same as that of humans, and the mass production of such humanoids will be possible. Further, the uncanny valley may disappear in humanoids. The present study indicates that clone faces elicit eeriness. This suggests an irony: even if this new technology can bridge the uncanny valley, a new eerie phenomenon (i.e., the clone devaluation effect) would appear alongside new technologies like human cloning techniques or robotic development. The results of a previous study that investigated public attitudes towards human cloning in the United Kingdom found that people have relatively negative responses to reproductive cloning technology [37]. However, unlike this survey, the present study created an experimental situation in which new technologies such as cloning human were realized and found that people tended to evaluate clone faces negatively. Therefore, our findings shed light on the negative aspects of the development of new technology and we urge the reconsideration of the rapid introduction of such technologies into society from the perspective of psychological responses instead of bioethics.

The present study has some limitations, which suggest the need for further research on the clone devaluation effect. First, we used dog faces in Study 3 to investigate the influence of distinguishableness of faces on the clone devaluation effect. However, there is another effect that enables human beings to easily distinguish faces that belong to their own racial group, which is known as the other-race effect [e.g., 38]. To explore the influence of distinguishableness in detail, this effect may be helpful. If other-race faces are used as clone faces, the clone devaluation effect would be weaker than own-race faces or not occur. Second, we should also set up an experimental scene to simulate real-life situations in the near future. In most of our studies, we presented clone faces of humans mainly because we assumed a future where technology overcomes the uncanny valley. However, it is possible that robots with features less similar to human faces, which have not fallen into the uncanny valley, will become popular before more human-like robots will be available on the market. As the results of Study 3 imply, the clone devaluation effect is unlikely to occur in non-human clone faces. Considering this, we speculated that if robots with features less similar to human faces are clearly perceived as robots, not humans, the clone devaluation effect would not occur in clone faces of such robots. Third, it is possible that the observation time of the clone faces varied depending on the participants, which may have affected the results of the present study since we did not control the exposure time of clone faces. Observing clone faces for a long time may lead to habituation, resulting in reduced clone devaluation effects. Therefore, to reveal a temporal aspect of the clone devaluation effect (e.g., minimum time for the clone devaluation effect to occur) future studies should manipulate the exposure time of clone faces.

In conclusion, the present study has identified the clone devaluation effect, which asserts that clone faces elicit eeriness. The improbability and lack of humanity of the clone faces were related to this effect. Moreover, the duplication of identity, not facial features, had key roles in the clone devaluation effect. Furthermore, the clone devaluation effect stemmed from disgust and avoidance reactions (particularly, animal-reminder disgust). The present study suggests it is possible that the introduction of new technology in robotics or the cloning of human beings into society may cause unpleasant psychological reactions in the future.

## Supporting information

S1 Text Information on the supplementary experiment. (DOCX)

## References

[1] Silver D, Huang A, Maddison CJ, Guez A, Sifre L, van den Driessche G, et al. Mastering the game of Go with deep neural networks and tree search. Nature. 2016;529: 484–489. doi: 10.1038/nature16961 26819042

[2] Tanaka F, Isshiki K, Takahashi F, Uekusa M, Sei R, Hayashi K. Pepper learns together with children: Development of an educational application. 2015 IEEE-RAS 15th International Conference on Humanoid Robots (Humanoids). ieeexplore.ieee.org; 2015. pp. 270–275. doi: 10.1109/HUMANOIDS.2015.7363546

[3] Mori M. Bukimi no tani [the uncanny valley]. Energy. 1970;7: 33–35.

[4] MacDorman KF, Ishiguro H. The uncanny advantage of using androids in cognitive and social science research. Interaction Studies. 2006;7: 297–337. Available: http://www.macdorman.com/kfm/writings/pubs/MacDorman2006AndroidScience.pdf

[5] Burleigh TJ, Schoenherr JR, Lacroix GL. Does the uncanny valley exist? An empirical test of the relationship between eeriness and the human likeness of digitally created faces. Comput Human Behav. 2013;29: 759–771. doi: 10.1016/j.chb.2012.11.021

[6] Nishio S, Ishiguro H, Hagita N. Geminoid: Teleoperated Android of an Existing Person. In: de Pina Filho AC, editor. Humanoid Robots. Rijeka: IntechOpen; 2007. doi: 10.5772/4876

[7] Sakamoto D, Kanda T, Ono T, Ishiguro H, Hagita N. Android as a telecommunication medium with a human-like presence. 2007 2nd ACM/IEEE International Conference on Human-Robot Interaction (HRI). ieeexplore.ieee.org; 2007. pp. 193–200. doi: 10.1145/1228716.1228743

[8] Saya. [cited 15 Jan 2021]. Available: https://www.telyuka.com/

[9] Pandey AK, Gelin R. A Mass-Produced Sociable Humanoid Robot: Pepper: The First Machine of Its Kind. IEEE Robot Autom Mag. 2018;25: 40–48. doi: 10.1109/MRA.2018.2833157

[10] Liu Z, Cai Y, Wang Y, Nie Y, Zhang C, Xu Y, et al. Cloning of Macaque Monkeys by Somatic Cell Nuclear Transfer. Cell. 2018;172: 881–887.e7. doi: 10.1016/j.cell.2018.01.020 29395327

[11] Willis J, Todorov A. First impressions: making up your mind after a 100-ms exposure to a face. Psychol Sci. 2006;17: 592–598. doi: 10.1111/j.1467-9280.2006.01750.x 16866745

[12] Oosterhof NN, Todorov A. The functional basis of face evaluation. Proc Natl Acad Sci U S A. 2008;105: 11087–11092. doi: 10.1073/pnas.0805664105 18685089 PMC2516255

[13] Walker D, Vul E. Hierarchical encoding makes individuals in a group seem more attractive. Psychol Sci. 2014;25: 230–235. doi: 10.1177/0956797613497969 24163333

[14] Pool E, Brosch T, Delplanque S, Sander D. Attentional bias for positive emotional stimuli: A meta-analytic investigation. Psychol Bull. 2016;142: 79–106. doi: 10.1037/bul0000026 26390266

[15] Yamada Y, Kawabe T, Ihaya K. Can you eat it? A link between categorization difficulty and food likability. Adv Cogn Psychol. 2012;8: 248–254. doi: 10.2478/v10053-008-0120-2 22956990 PMC3434679

[16] MacDorman KF, Chattopadhyay D. Reducing consistency in human realism increases the uncanny valley effect; increasing category uncertainty does not. Cognition. 2016;146: 190–205. doi: 10.1016/j.cognition.2015.09.019 26435049

[17] Kawabe T, Sasaki K, Ihaya K, Yamada Y. When categorization-based stranger avoidance explains the uncanny valley: A comment on MacDorman and Chattopadhyay (2016). Cognition. 2017. pp. 129–131. doi: 10.1016/j.cognition.2016.09.001 27642031

[18] Yamada Y, Kawabe T, Ihaya K. Categorization difficulty is associated with negative evaluation in the “uncanny valley” phenomenon. Jpn Psychol Res. 2013;55: 20–32. doi: 10.1111/j.1468-5884.2012.00538.x

[19] Alter AL, Oppenheimer DM. Uniting the Tribes of Fluency to Form a Metacognitive Nation. Pers Soc Psychol Rev. 2009;13: 219–235. doi: 10.1177/1088868309341564 19638628

[20] Zajonc RB. Attitudinal effects of mere exposure. J Pers Soc Psychol. 1968;9: 1–27. doi: 10.1037/h0025716 5667435

[21] Reber R, Winkielman P, Schwarz N. Effects of Perceptual Fluency on Affective Judgments. Psychol Sci. 1998;9: 45–48. doi: 10.1111/1467-9280.00008

[22] Oppenheimer DM, Meyvis T, Davidenko N. Instructional manipulation checks: Detecting satisficing to increase statistical power. J Exp Soc Psychol. 2009;45: 867–872. doi: 10.1016/j.jesp.2009.03.009

[23] Sasaki K, Ihaya K, Yamada Y. Avoidance of Novelty Contributes to the Uncanny Valley. Front Psychol. 2017;8: 1792. doi: 10.3389/fpsyg.2017.01792 29123490 PMC5662646

[24] Yamada Y, Sasaki K, Kunieda S, Wada Y. Scents boost preference for novel fruits. Appetite. 2014;81: 102–107. doi: 10.1016/j.appet.2014.06.006 24933686

[25] Robbins R, McKone E. No face-like processing for objects-of-expertise in three behavioural tasks. Cognition. 2007;103: 34–79. doi: 10.1016/j.cognition.2006.02.008 16616910

[26] Lakens D, Scheel AM, Isager PM. Equivalence Testing for Psychological Research: A Tutorial. Advances in Methods and Practices in Psychological Science. 2018;1: 259–269. doi: 10.1177/2515245918770963

[27] van Overveld WJM, de Jong PJ, Peters ML, Cavanagh K, Davey GCL. Disgust propensity and disgust sensitivity: Separate constructs that are differentially related to specific fears. Pers Individ Dif. 2006;41: 1241–1252. doi: 10.1016/j.paid.2006.04.021

[28] Iwasa K, Tanaka T, Yamada Y. Factor structure, reliability, and validity of the Japanese version of the Disgust Scale-Revised (DS-R-J). The Japanese Journal of Psychology. 2018;89: 82–92. doi: 10.4992/jjpsy.89.16230 PMC5061427 27732659

[29] Haidt J, McCauley C, Rozin P. Individual differences in sensitivity to disgust: A scale sampling seven domains of disgust elicitors. Pers Individ Dif. 1994;16: 701–713. doi: 10.1016/0191-8869(94)90212-7

[30] Rozin P, Haidt J. The domains of disgust and their origins: contrasting biological and cultural evolutionary accounts. Trends in cognitive sciences. 2013. pp. 367–368. doi: 10.1016/j.tics.2013.06.001 23773551

[31] MacDorman KF, Entezari SO. Individual differences predict sensitivity to the uncanny valley. Interact Stud. 2015;16: 141–172. doi: 10.1075/is.16.2.01mac

[32] Burton AM, Wilson S, Cowan M, Bruce V. Face Recognition in Poor-Quality Video: Evidence From Security Surveillance. Psychol Sci. 1999;10: 243–248. doi: 10.1111/1467-9280.00144

[33] O’Toole AJ, Phillips PJ, Weimer S, Roark DA, Ayyad J, Barwick R, et al. Recognizing people from dynamic and static faces and bodies: dissecting identity with a fusion approach. Vision Res. 2011;51: 74–83. doi: 10.1016/j.visres.2010.09.035 20969886

[34] Chatterjee A. Neuroaesthetics: a coming of age story. J Cogn Neurosci. 2011;23: 53–62. doi: 10.1162/jocn.2010.21457 20175677

[35] Haxby JV, Ida Gobbini M. Distributed Neural Systems for Face Perception. In: Rhodes G, Calder A, Johnson M, Haxby JV, editors. Oxford Handbook of Face Perception. Oxford University Press; 2011. pp. 99–110. doi: 10.1093/oxfordhb/9780199559053.013.0006

[36] Leder H, Belke B, Oeberst A, Augustin D. A model of aesthetic appreciation and aesthetic judgments. Br J Psychol. 2004;95: 489–508. doi: 10.1348/0007126042369811 15527534

[37] Shepherd R, Barnett J, Cooper H, Coyle A, Moran-Ellis J, Senior V, et al. Towards an understanding of British public attitudes concerning human cloning. Soc Sci Med. 2007;65: 377–392. doi: 10.1016/j.socscimed.2007.03.018 17449156

[38] Walker PM, Tanaka JW. An encoding advantage for own-race versus other-race faces. Perception. 2003;32: 1117–1125. doi: 10.1068/p5098 14651324

