# Supplementary material for: The clone devaluation effect: A new uncanny phenomenon concerning facial identity
Source: PLoS One. 2021 Jul 13;16(7):e0254396. doi: 10.1371/journal.pone.0254396 (PMC8277018; doi:10.1371/journal.pone.0254396)
Supplement: S1 Text — (DOCX) [file pone.0254396.s001.docx]

**S1 Text.**

We conducted a supplementary experiment with white people as participants to investigate whether the clone devaluation effect occurs in not only Japanese but also other ethnic groups. The following is detailed information on the methods and results of the experiment.

**Method**

Thirty-five white people were recruited and participated in the experiment online (10 women, mean age = 27.97). Stimuli, procedure, and data analysis were identical to Study 1.

**Results**

Fig S1 shows the means of the eeriness, valence, and realism scores. The ANOVA on the subjective eeriness scores revealed that the main effect of image conditions was significant (*F*(2, 68) = 7.4649, *p* < .01, η*_p_*^2^ = .15). Multiple comparisons showed that the eeriness of the clone condition was significantly higher than the non-clone condition and single condition (clone vs. non-clone: *t*(34) = 2.78, *p* < .01, Cohen’s *dz* = 0.44; clone vs. single: *t*(34) = 3.33, *p* < .01, Cohen’s *dz* = 0.83). However, there was no significant difference between the non-clone and single conditions (*t*(34) = 0.27, *p* = .79, Cohen’s *dz* = 0.06).

The ANOVA on the emotional valence scores revealed that the main effect of image conditions was significant (*F*(2, 68) = 8.91, *p* < .001, η*_p_*^2^ = .21). Multiple comparisons showed that the clone condition was significantly more negative than the non-clone condition and single condition (clone vs. non-clone: *t*(34) = 3.55, *p* < .01, Cohen’s *dz* = 0.60; clone vs. single: *t*(34) = 3.9212, *p* < .001, Cohen’s *dz* = 0.66). However, there was no significant difference between the non-clone and single conditions (*t*(34) = 1.56, *p* = .13, Cohen’s *dz* = 0.0).

The ANOVA on the realism scores revealed that the main effect of image conditions was significant (*F*(2, 68) = 7.4649, *p* < .01, η*_p_*^2^ = .18). Multiple comparisons showed that the realism of the clone condition was significantly lower than the non-clone condition and single condition (clone vs. non-clone: *t*(34) = 2.62, *p* < .05, Cohen’s *dz* = 0.44; clone vs. single: *t*(34) = 4.89, *p* < .001, Cohen’s *dz* = 0.83). However, there was no significant difference between the non-clone and single conditions (*t*(34) = 0.33, *p* = .74, Cohen’s *dz* = 0.06).

**Fig S1. The results of the eeriness, valence, and realism evaluation in the supplementary experiment.** Error bars indicate the standard errors of the mean. The vertical axes indicate the mean eeriness (A), valence (B), realism scores (C) for images in each condition. The lower scores indicate more negative and improbable evaluation.
